# Nurses’ Occupational and Medical Risks Factors of Leaving the Profession in Nursing Homes

**DOI:** 10.3390/ijerph15091850

**Published:** 2018-08-27

**Authors:** Carole Pélissier, Barbara Charbotel, Jean Baptiste Fassier, Emmanuel Fort, Luc Fontana

**Affiliations:** 1 Unité Mixte de Recherche Épidémiologique et de Surveillance Transport Travail Environnement (UMRESTTE, UMR_T9405), Institut français des sciences et technologies des transports, de l’aménagement et des réseaux (IFSTTAR), Université de Lyon, Université Lyon 1, Université de St Etienne, 42055 Saint-Etienne, France; luc.fontana@chu-st-etienne.fr; 2 Service de Santé au Travail et Pathologies Professionnelles, CHU de Saint-Etienne, 42055 Saint-Etienne, France; 3 Unité Mixte de Recherche Épidémiologique et de Surveillance Transport Travail Environnement (UMRESTTE, UMR_T9405), Institut français des sciences et technologies des transports, de l’aménagement et des réseaux (IFSTTAR), Université de Lyon, Université Lyon 1, F-69373 Lyon, France; barbara.charbotel-coing-boyat@univ-lyon1.fr (B.C.); jean-baptiste.fassier@chu-lyon.fr (J.B.F.); emmanuel.fort@univ-lyon1.fr (E.F.); 4 Hospices Civils de Lyon, Centre Hospitalier Lyon Sud, Service de Pathologies Professionnelles, F-69495 Pierre Bénite, France; 5 Hospices Civils de Lyon, Service de Médecine et Santé au Travail, 69002 Lyon, France

**Keywords:** care workers’ intention to leave, nursing homes for the elderly, psychosocial factors, musculoskeletal complaints, impaired well-being

## Abstract

This study aimed to evaluate the association between intention to leave work, and working conditions and health status among female care-staff in nursing homes. A multicenter cross-sectional study included female care-staff in 105 nursing homes for the elderly. We used validated questionnaires to assess occupational, psychosocial and medical data in a multicenter transverse study. Univariate analysis on chi² test was performed with stratification according to job (nurse, nursing assistant), and variables found to be significant on each dimension were included on multivariate models. 1428 nursing assistants and 342 registered nurses were included. 391 nursing assistants and 85 registered nurses intended to leave their work with the elderly. The registered nurses’ intention to leave was associated with deteriorated care-team or residents relations, and with perceived elevated hardship due to the proximity of residents’ death. The nursing assistants’ intention to leave was associated with deteriorated management relation, with job insecurity and elevated hardship due to the residents’ intellectual deterioration. Impaired physical or psychological health status also correlated with this intention. Policy to reduce voluntary turnover of care-staff in nursing homes for the elderly could be based on multifactorial management, acting on work organization and reducing psychosocial stress.

## 1. Introduction

High rates of staff turnover in nursing homes are not a recent phenomenon, especially among those who work most closely with the residents: registered nurses and nursing assistants. Nursing homes for the elderly differ in functioning from hospitals in that they have lower level of medical presence. They are residential establishments caring for increasingly elderly, dependent persons with multiple comorbidities. Care is given by nursing assistants with various kinds of initial training and by registered nurses. Registered nurses usually ensure technical care and coordinate the work of the nursing assistants. As well as catering and accompaniment, nursing assistants are in charge of hygiene, comfort and preventive and curative care and are under the supervision of a nurse. About 17.5% of new nurses leave their first job within one year of starting their jobs [1]. The proportion of nurses considering or intending to leave the profession varied from 4% to 54% [2]. The cost of turnover from the organizational perspective includes replacement costs (including training), lost productivity, lost quality and lowered morale [3,4]. A previous study argued that these costs may well be reflected in the quality of care that residents receive [3,5]. Three broad categories of factors that influence turnover have been identified: general economic, work-related and personal factors [3]. Registered nurses and nursing assistants in nursing homes are subject to strong mental and physical demands (e.g., lifting and carrying, work schedule), and frequently describe their working environment as distressing [6]. The physical and mental deterioration of elderly patients and the proximity to death were more often perceived as a source of hardship by registered nurses and nursing assistants. Registered nurses and nursing assistants are more often confronted by aggressive patients than in hospitals or facilities for the disabled [7,8,9]. Registered nurses who had experienced burnout or stress were more likely to intend to leave the profession [2,10]. Fochsen et al. have focused on the relationship between musculoskeletal disorders and intent to leave the profession in staff of nursing-homes for the elderly [11]. Several studies underlined an association between impaired mental well-being in nursing home care workers and psychosocial factors in health caregivers, but there has been less research on the association between impaired mental well-being in nursing home care workers and health caregivers’ intention to leave has hardly been studied [12,13,14,15]. Understanding why health care workers abandon their current employer and/or their job in the nursing profession is important in retention of health providers. The objective of the present study was to analyze occupational and medical factors associated with the intention to leave work with the elderly, comparing between nursing assistants and registered nurses, considering the differences in their tasks.

## 2. Materials and Methods

### 2.1. Design

The study was designed as a cross-sectional questionnaire survey design.

### 2.2. Participants

The target population of the survey was female employees working in with elderly patients in nursing homes in the Rhône-Alpes Region of France. The occupational physicians of the Region were asked to participate in the survey by the Regional Department of Businesses, Competition, Consumption, Work and Employment (DIRECCTE), a state business consultancy. Volunteer occupational physicians filled out a working conditions assessment questionnaire, and asked all employees meeting the inclusion criteria in the nursing homes which they oversaw to take part. Only employees who had been working with the elderly for at least 6 months on at least a half-time basis were included. The occupational physician handed over the self-administered questionnaire to subjects included in the study and collected data on working conditions and health status in nursing staff. The number of subjects who refused to participate was anonymously collected by the occupational physicians.

### 2.3. Data Collection

Between October 2009 and September 2010, data were collected by self-administered questionnaires during periodic follow-up medical examinations or during company visits. These self-administered questionnaires comprised personal characteristics (age, gender, family status, number of children), health characteristics and work-related characteristics and used four questionnaires measuring Job related hardship, musculoskeletal complaints, impaired mental well-being, and psychosocial demands [16,17,18,19,20,21].

### 2.4. Measures

*Job related hardship*: The questionnaire included visual analog hardship scales (1 = no hardship, to 10 = great hardship) related to: premises (disrepair, stairs, clutter), lifting and carrying the elderly, patients’ mental and physical deterioration, and proximity of death [16,17,18]. The choice of these particular categories was based on the literature [21]. Hardship scores were categorized as slight (≤3), moderate (4–7) or severe hardship (≥8).

*Musculoskeletal complaints*: Musculoskeletal complaints were assessed on the Nordic Musculoskeletal Questionnaire, comprising multiple choice questions for each body part (such as upper limbs, lower limbs, neck and back): “During the last 12 months, have you had trouble (such as ache, pain, discomfort)?”. The Nordic Musculoskeletal Questionnaire is a standardized questionnaire with reliability and validity moderate to high, which makes is a good instrument for epidemiological survey into musculoskeletal disorders and complaints [19].

*Impaired mental well-being*: Developed as a screening tool to detect the workers likely to have or to risk of developing psychiatry disorders, the General Health Questionnaire (GHQ) is a measure of the common mental health problems, domains of depression, anxiety, somatic symptoms and social withdrawal. It’s the 12-item version (GHQ-12) was used. Respondents had to indicate how frequently they had recently experienced the various symptoms listed. Each item was rated on a 4-point scale, with weights from 0 to 3 according to Likert-like type scale (0 = less than usual, 1 = no more than usual, 2 = rather more than usual, and 3 = much more than usual) [20]. Scores higher than 12 were considered as indicating impaired mental wellbeing [22,23].

*Psychosocial demands*: Psychosocial demands at work were assessed on the effort-reward imbalance (ERI) model using the Siegrist questionnaire. In this model, chronic work-related stress is identified as non-reciprocity or imbalance between high efforts spent and low rewards received. Conversely, positive emotions evoked by appropriate social rewards promote well-being, health and survival [16]. This questionnaire has three scales: two measuring the extrinsic components of ‘effort’ (6 items) and ‘reward’ (11 items covering earnings, esteem and job security) and one measuring the intrinsic component of ‘over-commitment’ (six items) [16,17]. Effort was measured either by six items on the demanding aspect of the work environment (three measuring quantitative load, one qualitative load, one increase in total load over time, and one physical load), rated as 1 = does not apply, 2 = does apply but subject does not consider herself distressed, 3 = does apply and subject considers herself somewhat distressed, 4 = does apply and subject considers herself distressed, or 5 = does apply and subject considers herself very distressed. A sum score of these ratings was totalled, as documented in several studies [16,17]. According to the ERI model, extrinsic and intrinsic effort scores are directly proportional to effort, whereas the rewards score is inversely proportional to reward. ERI was measured by calculating the ratio between the extrinsic effort index (E) and the inverse reward index (R): E/(R*c), with c as a correction factor (c: 6/11); ERI >1 indicates a critical condition of high-cost/low-gain, or effort-reward imbalance [16,17]. To obtain a more precise view of which rewards were related to which indicators of employee health, three occupational rewards (earnings, esteem and job security) were assessed separately. All three reward scores were dichotomized separately, using the most adverse tercile to indicate low reward: the higher the reward score, the lower the reward [16,17,18,21,24,25].

### 2.5. Ethical Considerations

Approval by the French Ministry of Health Research (Comité Consultatif pour le Traitement de l’Information en Matière de Recherche dans le Domaine de la Santé, 09.320) was obtained before starting the study. The participants were given an information leaflet explaining the study objectives and participation was voluntary.

### 2.6. Data Analysis

The continuous quantitative variable of seniority in the establishment was transformed into an ordinal qualitative variable for statistical purposes.

A descriptive step characterized the employee population according to personal characteristics, working conditions, including occupational psychosocial factors, and mental health status. Distinguishing between registered nurses and nursing assistants, associations were sought between the intent to leave work with the elderly and personal factor, occupational factors, psychosocial factors and medical factors. Frequencies were compared on chi-squared tests, with chi-squared trend tests depending on the results of cross-analysis. Ratios of event probabilities per case of intent to leave were studied. As the prevalence of each event was high, odds ratios would not provide a good estimate of relative risk, and a log-linked binomial model was applied, using the PROC GENMOD procedure in the SAS statistical package (version 9.3) with the DIST = BINOMIAL and LINK = LOG options.

Univariate and multivariate analyses were performed stratified on job category (nurse/nursing assistant). The binary response of each case of intent to leave was modeled in two steps: Firstly, all independent variables underwent univariate analysis; secondly, multivariate analyses were performed for each dimension (sociodemographic, occupational, psychosocial, and personal health); variables with *p*-value ≤ 0.1 were included in a multivariate model on a descending procedure, and variables with *p*-value < 0.05 were kept in the model. The psychosocial and medical dimension models were adjusted on age. For the psychosocial dimension, the global ERI variable is classically used, but in the present study we sought to investigate the questionnaire sub-dimensions in greater detail, studying the four variables of extrinsic effort and the three types of reward (esteem, earnings, and job security) in the multivariate models. From these four multivariate models, multivariate regression was performed on a descending procedure, with a significance threshold of 5%. In case of non-convergence of PROC GENMOD because the maximum likelihood estimate (MLE) lay on the boundary of the parameter space, the SAS COPY macro was used, which provides a good approximation to the exact maximum likelihood estimates, as well as yielding good estimates of the true population parameters [26].

## 3. Results

### 3.1. Socio-Occupational and Medical Data

1770 women (19.6% registered nurses, 80.4% nursing assistants) working in direct contact with the elderly in 105 nursing homes were included. Twenty-nine registered nurses and nine nursing assistants refused to participate, leading to a global participation rate of 98%. Four hundred and seventy-six of the 1808 women (26.3%), wished to leave their work with the elderly: 391 nursing assistants (26.8%) and 85 registered nurses (24.2%).

### 3.2. Factors Associated with Intention to Leave Work on Univariate Analysis

Occupational factors: In both nursing assistants and registered nurses, severe hardship related to residents’ physical deterioration, to proximity to death, or to lack of equipment was significantly associated with intention to leave. In nursing assistants, weak attachment to the elderly residents, understaffing, and severe hardship related to lifting and carrying residents, to premises, or to residents’ intellectual deterioration, were significantly associated with intention to leave (Table 1). In both nursing assistants and registered nurses, work contract, seniority in work with elderly, working time, night work and type of working hours were not associated with intention to leave.

*Psychosocial factors*: In both nursing assistants and registered nurses, intention to leave was significantly associated with impaired relations with residents, poor reward in terms of earnings, job security, and esteem, over-commitment, and ERI.

In nursing assistants, intention to leave was significantly associated with physical, or verbal aggression, and impaired relations with management, or families.

In registered nurses, intention to leave was significantly associated with impaired relations with the care-team (Table 1).

*Med Psychosocial factors*: In both nursing assistants and registered nurses, intention to leave was significantly associated with impaired relations with residents, poor reward in terms of earnings, job security, and esteem, over-commitment, and ERI.

In nursing assistants, intention to leave was significantly associated with physical, or verbal aggression, and impaired relations with management, or families.

In registered nurses, intention to leave was significantly associated with impaired relations with the care-team (Table 1).

*ical factors*: For both nursing assistants and registered nurses, psychological distress and spinal, or lower-limb complaints were significant. Upper-limb musculoskeletal disorder was significantly associated with intention to leave in nursing assistants (Table 1).

### 3.3. Factors Associated with Intention to Leave Work on Multivariate Analysis

*Occupational factors*: Severe hardship related to proximity to death was associated with intention to leave in registered nurses, as was severe hardship related to residents’ intellectual deterioration and weak attachment to residents in nursing assistants (Table 2, Table 3 and Table 4). In this global multivariate analysis, hardship related to inadequate equipment no longer featured as a significant factor (Table 4).

*Psychosocial factors*: Impaired relations with management in nursing assistants, impaired relations with residents or with care team in registered nurses, remained associated with intention to leave. In nursing assistants, high levels of effort and poor reward in terms of job security were associated with intention to leave. Poor esteem from colleagues no longer featured as a significant factor on global multivariate analysis (Table 2 and Table 4).

*Medical factors*: Lower limb complaints and spinal complaints remained associated with intention to leave in nursing assistants but not registered nurses on global multivariate analysis (Table 4).

## 4. Discussion

In the present study, care-workers’ intention to leave was significantly related to certain psychosocial factors and impaired health status. This study of 1770 female registered nurses and nursing assistants in 105 nursing homes for the elderly in the Rhône-Alpes Region of France had a 98% response rate. This study has the advantage of having exploited for the administration of the questionnaires the periodic visits conducted by the occupational physicians. This method usually allows a high participation and minimizes the possibility that the answers are influenced by other workers, because the time in the waiting room is very limited [27].

Validated questionnaires assessed psychosocial demand, musculoskeletal complaints and psychological distress. The interest of the study lay in correlating intention to leave work with the elderly and working conditions (psychosocial and organizational demand) while taking into account the registered nurses’ and nursing assistants’ psychological and physical health status. This cross-sectional study does not allow us to know the temporal relation between the different phenomena that influence the intention to leave. However, previous studies have made it clear that the various factors, violence and stress, are cyclically related to each other [28,29,30].

Four hundred and seventy-six (26.3%) of respondents wished to leave their work with the elderly, and nursing assistants (26.8%) more frequently than registered nurses (24.2%). This finding agrees with Fochsen et al., who reported that “being an assistant nurse was associated with leaving nursing care” [11]. The NEXT-Study (Nurses’ early exit study) was established to investigate the degree, reasons, circumstances, and consequences of premature departure from the nursing profession in Europe. The proportion of nurses (24.9%) who had frequently considered leaving their profession in nursing homes was higher in our study than in the French data of the longitudinal NEXT-Study (16.5%), in which nurses were working in residential care for the elderly, hospital structures or home-care [31]. The negative relation-ship between age and turnover intent has been widely reported in previous studies [13,32]. It has been argued that young nurses who belong to generation Y (born after 1980) hold different attitudes and values towards their work, which may influence their retention. According to Lavoie-Tremblay et al., the generation Y nurses reported that recognition was a key motivator. Their needs are stability, flexible work schedules and shifts, recognition, opportunities for professional development, and adequate supervision [33].

The study highlighted a significant association between hardship in relation to proximity to death and intention to leave work with the elderly. Previous study showed that nursing home staff who had had palliative care training were less liable to report severe hardship in relation to proximity to death, suggesting that palliative care training has positive impact on perceived hardship in relation to proximity to death [34]. Better training in the management of dementia patients and in palliative care might reduce the intention to leave work with the elderly.

A number of researchers have reported an association between workload, stress and turnover intention [35,36]. In this cross-sectional study, both workplace violence and bad relationships (which often fall into verbal violence) are significantly associated with the intention to leave work. The effect was higher in younger workers; this confirms what observed in previous Italian cohorts of nurse and nursing students [37]. Previous studies suggested that the psychologically strenuous and stressful nature of the work causes nurses to consider leaving the nursing profession [38,39,40]. In the present study, intention to leave was significantly related to certain psychosocial factors. First effort/reward imbalance was significantly related to intention to leave in nursing assistants. The ERI model was used to analyze intention to leave the nursing profession in nurses (*n* = 21,299) in seven European countries, in the NEXT-Study: ERI was associated with the frequency of considering leaving the nursing profession [31]. Secondly, overcommitment was not significantly associated with intention to leave in the present study, in agreement with previous findings [31,40]. According to Zeytinoglu et al., nurses with higher levels of career commitment showed statistically significantly lower levels of propensity to leave nursing [10]. Wang et al. found that a higher level of occupational commitment was related to stronger intention to stay [41]. Low affective commitment to the profession was associated with greater intention to leave the profession [2]. Moreover, Takase et al. found that nursing staff commitment was directly related to reduce intention to quit the profession [42]. The present study highlighted weak attachment to residents on the part of nurses and nursing assistants as associated with intention to leave. Then high levels of effort were an important work environment factor for intention to leave, consistent with previous findings [31,32,33,34,35,36,37,38,39,40]. Finally, for nursing assistants, poor reward in terms of job security, but in terms of not earnings, was significantly associated with intention to leave on multivariate analysis. Pay was found not a significant predictor for Jordanian nurses to leave their organization [43]. According to Lavoie-Tremblay et al., the reasons most often given for quitting the profession by nurses were difficult working conditions and a lack of job stability [34]. Improving the psychosocial work environment and specifically occupational rewards may be helpful in retaining caregivers.

Poor relations with management for nursing assistants and poor relations with the care-team and patients for nurses were significantly associated with intention to leave [44]. Nursing assistants seemed to exchange little with nurses according to an ergonomic study of geriatric care-staff [45]. In France, the working week was reduced to 35 h, which may have had the effect of reducing the time available for verbal exchanges within the team. Moreover, increasing administrative work for nurses and the increased work-load reported may have reduced the time available for verbal exchanges between patients and nurses, impairing the nurse-patient relationship. Cowden et al., in a systematic review of the literature, found a positive relationship between transformational leadership, supportive work environments and staff nurses intentions to stay [46]. Creating a supportive work environment, with supportive leadership gives nurses the confidence to stay [47,48]. Healthy and supportive work relationships have been shown to be related to greater intention to stay in work for nurses [49,50].

The present study revealed a significant association between intention to leave and impaired health status. First, lower-limb and spinal musculoskeletal complaints were significantly associated with intention to leave for nursing assistants. Fochsen and Sjögren showed that musculoskeletal problems of the neck/shoulder and knees were associated with leaving nursing care [11,51]. Secondly, psychological distress was significantly associated with intention to leave for both nursing assistants and nurses, in line with the longitudinal study of 6441 caregivers by Kivimaki et al. [52]. Bartram et al. found that emotional labor has an indirect effect on intention to leave via burnout nurses experience [53].

## 5. Conclusions

Care-staff in nursing homes for the elderly are exposed to elevated psychological and physical demands in caring for dependent elderly persons often with comorbidities. The present study found that some of the demands experienced by nurses and nursing assistants underlie an intention to leave work with the elderly. The results highlight the importance of perceived working conditions in this intention to leave. Policy to reduce voluntary turnover in nursing homes for the elderly could be based on a multifactorial approach involving work organization, reduced psychosocial demand and access to certain training modules such as in palliative care and the management of patients in dementia. Reorganization to reduce nurses’ administrative workload by delegating tasks to administration workers should allow nurses more time for exchanges with both patients and nursing assistants. Having more time to devote to relational aspects would enhance verbal exchange between residents and caregivers, which in turn should improve job satisfaction and quality of care. Greater involvement in drawing up personalized care projects and reinforcing and developing caregiver autonomy in performing everyday care tasks should improve the caregivers’ image of the job. Better recognition of the work accomplished could be achieved by increasing the time available for exchanges between colleagues and with management, and by improving job security.

## Figures and Tables

**Table 1:** Sociodemographic, occupational, psychosocial and medical factors associated with registered nurses’ and nursing assistants’ intention to leave work with the elderly, on univariate analysis.

		Nursing Assistants’ Intention to Leave (*n* = 391)				Registered Nurses’ Intention to Leave (N = 85)			
**Sociodemographic Factors**		**N**	**%**	**PR**	**[95% CI]**	**N**	**%**	**PR**	**[95% CI]**
Age	<30 years	118	33.9	1 ****	17	27.9	1 ^∇^	
	30–39 years	97	31.0	0.91	0.74–1.14	24	33.3	1.19	0.71–2.01
	40–49 years	110	24.5	0.72	0.58–0.90	24	25.8	0.93	0.54–1.57
	≥50 years	65	20.5	0.60	0.46–0.78	20	17.2	0.62	0.35–1.09
Marital status	Single	92	32.7	1 *	10	29.4	1	
	In couple	237	25.4	0.77	0.64–0.94	62	24.8	0.84	0.48–1.48
	Separated, divorced, widowed	58	28.6	0.87	0.66–1.15	13	22.8	0.77	0.38–1.57
Do you have children?	Yes	267	25.4	1 **	68	24.9	1	
	No	123	33.0	1.30	1.09–1.55	17	24.6	0.98	0.62–1.57
**Occupational Factors**		**N**	**%**	**PR**	**[95% CI]**	**N**	**%**	**PR**	**[95% CI]**
Hardship: lifting and carrying	Mild	36	16.4	1 ****	18	20.4	1	
	Moderate	108	22.6	1.37	0.97–1.93	34	25.0	1.22	0.74–2.02
	Severe	245	34.1	2.07	1.51–2.85	32	28.6	1.40	0.84–2.31
Hardship: premises	Mild	161	11.4	1 **	28	20.3	1	
	Moderate	133	30.7	1.31	1.08–1.59	27	26.0	1.28	0.80–2.03
	Severe	245	32.7	1.40	1.13–1.73	30	31.60	1.56	0.99–2.42
Hardship: equipment	Mild	139	21.3	1 ****	32	20.0	1 *	
	Moderate	143	30.7	1.44	1.18–1.76	27	25.5	1.27	0.81–1.99
	Severe	103	36.9	1.73	1.39–2.14	25	36.2	1.81	1.16–2.81
Hardship: residents’ intellectual deterioration	Mild	54	16.8	1 ****	14	15.4	1	
	Moderate	115	25.1	1.44	1.18–1.76	28	21.1	1.57	0.88–2.80
	Severe	218	34.4	1.73	1.40–2.14	43	32.6	2.11	1.24–3.64
Hardship: residents’ physical deterioration	Mild	46	17.1	1 ****		13	15.7	1 *	
	Moderate	112	24.7	1.45	1.06–1.96	30	23.3	1.48	0.82–2.68
	Severe	230	33.1	1.94	1.46–2.57	42	38.8	2.09	1.20–3.66
Hardship: proximity to death	Mild	90	22.4	1 **		24	19.7	1 *	
	Moderate	128	27.0	1.21	0.95–1.52	32	22.7	1.15	0.72–1.85
	Severe	172	31.7	1.41	1.13–1.76	29	37.7	1.91	1.21–3.03
Do you get attached to the residents ?	Very often	59	21.4	1 *		6	13.0	1 ^∇^	
	Often	235	27.5	1.29	1.00–1.65	52	24.9	1.91	0.87–4.17
	Seldom	78	31.8	1.49	1.11–1.99	24	34.3	2.63	1.16–5.93
	Rarely/never	19	39.6	1.85	1.22–2.81	3	20.00	1.53	0.44–5.39
Staffing levels adequate?	Yes	58	20.1	1 ***		10	15.6	1 ^∇^	
	No	331	29.4	1.46	1.14–1.87	75	27.4	1.75	0.96–3.19
**Psychosocial Factors**		**N**	**%**	**PR**	**[95% CI]**	**N**	**%**	**PR**	**[95% CI]**
Victim of verbal aggression	No	51	17.6	1 ****		12	23.5	1	
	Yes	340	29.8	1.69	1.29–2.20	73	25.1	1.06	0.62–1.82
Victim of physical aggression	No	137	22.2	1 ****		41	23.7	1	
	Yes	254	31.3	1.41	1.18–1.69	44	26.0	1.09	0.76–1.59
Relations with team	Satisfactory/very satisfactory	308	26.5	1 ^∇^		60	21.1	1 ***	
	Moderately satisfactory/unsatisfactory	81	31.6	1.19	0.98–1.46	25	43.9	2.07	1.43–3.00
Relations with patients	Satisfactory/very satisfactory	339	26.0	1 ***		66	22.1	1 **	
	Moderately satisfactory/unsatisfactory	52	42.3	1.63	1.29–2.04	19	45.2	2.05	1.38–3.04
Relations with families	Satisfactory/very satisfactory	287	25.5	1 ****		58	24.1	1	
	Moderately satisfactory/unsatisfactory	76	40.6	1.59	1.30–1.95	27	28.4	1.18	0.79–1.74
Relations with management	Satisfactory/very satisfactory	231	22.4	1 ****		52	22.4	1	
	Moderately satisfactory/unsatisfactory	153	40.4	1.80	1.52–2.12	33	30.6	1.36	0.94–1.98
Effort/reward imbalance	No	317	25.0	1 ****		72	23.2	1 *	
	Yes	70	48.3	1.93	1.59–2.34	13	43.3	1.87	1.18–2.95
Overcommitment	No	268	25.2	1 **		48	21.0	1 *	
	Yes	123	33.7	1. 34	1.12–1.60	37	32.7	1.56	1.08–2.25
Extrinsic effort	No	243	22.6	1 ****		49	22.6	1	
	Yes	148	41.8	1.85	1.57–2.18	36	28.8	1.27	0.88–1.85
Reward	Yes	234	22.3	1 ****		60	22.2	1 *	
	No	157	41.6	1.87	1.59–2.20	25	34.7	1.56	1.06–2.30
Earnings	Yes	151	20.8	1 ****		45	20.9	1 *	
	No	226	35.8	1.72	1.44–2.05	37	33.0	1.57	1.09–2.28
Esteem	Yes	174	20.9	1 ****		41	20.3	1 **	
	No	202	37.6	1.80	1.51–2.13	43	35.6	1.65	1.15–2.39
Job security	Yes	128	19.1	1 ****		31	16.6	1 ****	
	No	258	35.9	1.89	1.57–2.26	54	35.8	2.15	1.46–3.17
**Medical Factors**		**N**	**%**	**PR**	**[95% CI]**	**N**	**%**	**PR**	**[95% CI]**
Psychological distress	No	200	37.8	1 ****		45	31.5	1 *	
	Yes	189	21.5	1.76	1.49–2.08	39	19.7	1.59	1.10–2.32
Spinal complaints	No	98	19.6	1 ***		22	17.9	1 *	
	Yes	293	31.7	1.62	1.32–1.98	61	28.2	1.58	1.02–2.44
Upper-limb complaints	No	303	26.2	1 *		63	22.9	1 ^∇^	
	Yes	85	33.5	1.28	1.05–1.56	21	33.3	1.45	0.96–2.19
Lower-limb complaints	No	311	25.8	1 ***		64	22.8	1 *	
	Yes	77	37.6	1.45	1.19–1.78	21	36.8	1.62	1.08–2.42

PR: Prevalence Ratio; CI: Confidence Interval. ^∇^ *p*-value < 0.1; * *p* < 0.05; ** *p* < 0.01; *** *p* < 0.001; **** *p* ≤ 10^−4^.

**Table 2:** Significant associations between nursing assistants’ intention to leave work with the elderly and sociodemographic, occupational, psychosocial and medical factors on intermediate multivariate analysis.

Variables		PR	[95% CI]
**Sociodemographic Factors**			
Age	<30 years	1 ***	
	30–39 years	0.91	0.74–1.14
	40–49 years	0.72	0.58–0.90
	≥50 years	0.60	0.46–0.78
**Occupational Factors**			
Work contract	Permanent	1 *	
	Temporary	0.78	0.63–0.97
Hardship: equipment	Mild	1 **	
	Moderate	1.23	1.00–1.51
	Severe	1.45	1.17–1.79
Hardship: residents’ intellectual deterioration	Mild	1 ****	
	Moderate	1.41	1.05–1.89
	Severe	1.94	1.48–2.55
Do you get attached to the residents?	Very often	1 **	
	Often	1.29	1.00–1.66
	Seldom	1.48	1.11–1.97
	Rarely/never	1.96	1.33–2.87
**Psychosocial Factors**			
Relations with management	Satisfactory/very satisfactory	1 *	
	Moderately satisfactory/unsatisfactory	1.26	1.04–1.55
Extrinsic effort	No	1 **	
	Yes	1.31	1.08–1.60
Esteem	Yes	1 *	
	No	1.26	1.01–1.57
Job security	Yes	1 ***	
	No	1.51	1.21–1.89
**Medical Factors**			
Psychological distress	No	1 ****	
	Yes	1.55	1.31–1.84
Spinal complaints	No	1 **	
	Yes	1.38	1.13–1.70
Lower-limb complaints	No	1 ***	
	Yes	1.39	1.15–1.69

PR: Prevalence Ratio; CI: Confidence Interval.* *p* < 0.05; ** *p* < 0.01; *** *p* < 0.001; **** *p* ≤ 10^−4^.

**Table 3:** Significant associations between registered nurses’ intention to leave work with the elderly and sociodemographic, occupational, psychosocial and medical factors on intermediate multivariate analysis.

Variables		PR	[95% CI]
**Sociodemographic Factors**			
Age	<30 years	1 *	
	30–39 years	1.20	0.71–2.01
	40–49 years	0.93	0.54–1.57
	≥50 years	0.62	0.35–1.10
**Occupational Factors**			
Hardship: equipment	Mild	1 *	
	Moderate	1.25	0.80–1.95
	Severe	1.75	1.14–2.68
Hardship: proximity to death	Mild	1 ***	
	Moderate	1.17	0.73–1.89
	Severe	1.95	1.23–3.08
**Psychosocial Factors**			
Relations with team	Satisfactory/very satisfactory	1 **	
	Moderately satisfactory/unsatisfactory	1.98	1.32–2.98
Relations with patients	Satisfactory/very satisfactory	1 *	
	Moderately satisfactory/unsatisfactory	1.43	0.93–2.19
**Medical Factors**			
Psychological distress	No	1 **	
	Yes	1.66	1.14–2.39
Spinal complaints	No	1 *	
	Yes	1.65	1.07–2.53

PR: Prevalence Ratio; CI: Confidence Interval. * *p* < 0.05; ** *p* < 0.01; *** *p* < 0.001.

**Table 4:** Significant associations between registered nurses’ and nursing assistants’ intention to leave work with the elderly and sociodemographic, occupational, psychosocial and medical factors on global multivariate analysis.

Nursing Assistants				Registered Nurses			
Variables		PR	[95% CI]	Variables		PR	[95% CI]
**Sociodemographic Factors**				**Sociodemographic Factors**			
Age	<30 years	1 ***		Age	<30 years	1 *	
	30–40 years	0.92	0.75–1.12		30–40 years	0.97	0.60–1.57
	40–50 years	0.74	0.61–0.90		40–50 years	0.80	0.49–1.33
	>50 years	0.58	0.46–0.74		>50 years	0.53	0.30–0.92
**Occupational Factors**				**Occupational Factors**			
Hardship: residents’ intellectual deterioration	Mild	1 ****		Hardship: proximity to death	Mild	1 *	
	Moderate	1.38	1.04–1.83		Moderate	1.23	0.79–1.93
	Severe	1.77	1.36–2.30		Severe	1.82	1.18–2.82
				**Psychosocial Factors**			
Do you get attached to the residents?	Very often	1 **		Relations with team	Satisfactory/very satisfactory	1 **	
	Often	1.28	1.01–1.63		Moderately satisfactory/unsatisfactory	1.71	1.15–2.56
	Seldom	1.33	1.02–1.73	Relations with patients	Satisfactory/very satisfactory	1 *	
	Rarely/never	1.96	1.63–2.82		Moderately satisfactory/unsatisfactory	1.52	1.02–2.26
**Psychosocial Factors**							
Relations with management	Satisfactory/very satisfactory	1 ***					
	Moderately satisfactory/unsatisfactory	1.34	1.14–1.58				
Extrinsic effort	No	1 *					
	Yes	1.22	1.02–1.45				
Job security	Yes	1 ***					
	No	1.42	1.17–1.72				
**Medical Factors**							
Spinal complaints	No	1 *					
	Yes	1.27	1.04–1.55				
Lower-limb complaints	No	1 **					
	Yes	1.34	1.13–1.59				

PR: Prevalence Ratio; CI: Confidence Interval. * *p* < 0.05; ** *p* < 0.01; *** *p* < 0.001; **** *p* ≤ 10^−4^.
